# Depression, substance abuse and stigma among men who have sex with men in coastal Kenya

**DOI:** 10.1097/QAD.0000000000000846

**Published:** 2015-12

**Authors:** Andrew M. Secor, Elizabeth Wahome, Murugi Micheni, Deepa Rao, Jane M. Simoni, Eduard J. Sanders, Susan M. Graham

**Affiliations:** a Department of Global Health, University of Washington, Seattle, Washington, USA; b Kenya Medical Research Institute-Wellcome Trust Research Programme, Kilifi, Kenya; c Department of Psychology, University of Washington, Seattle, Washington, USA; d Nuffield Department of Medicine, Centre for Tropical Medicine and Global Health, University of Oxford, Headington, UK; e Department of Global Health, Academic Medical Centre, University of Amsterdam, Amsterdam, The Netherlands; f Department of Medicine, University of Washington, Seattle, Washington, USA; g Department of Epidemiology, University of Washington, Seattle, Washington, USA

**Keywords:** depression, HIV/AIDS, homosexuality, Kenya, male, mental health, psychosocial, social stigma, substance-related disorders

## Abstract

**Objectives:**

Mental health conditions can erode quality of life and interfere with health-related behaviours such as medication adherence. We aimed to determine the prevalence and correlates of depression and other psychosocial factors among self-identified men who have sex with men (MSM) in coastal Kenya.

**Design:**

A cross-sectional survey.

**Methods:**

Psychosocial and mental health characteristics were assessed in an audio computer-assisted self-interview (ACASI) survey among 112 MSM participating in two ongoing HIV-positive and HIV-negative cohorts in Mtwapa, Kenya.

**Results:**

One-third of participants met criteria for major depressive disorder [16.1%, 95% confidence interval (95% CI) 9.8–24.2] or other depressive disorder (15.2%, 95% CI 9.1–23.2). Alcohol abuse was reported by 45% of respondents (95% CI 35.2–54.3) and other substance abuse by 59.8% (95% CI 50.1–69.0). Sexual and HIV stigma were moderate, with median scores of 11 [interquartile range (IQR) 6–17, potential range 0–33] and 25 (IQR 23–29, potential range 11–44), respectively. There were significant bivariate correlations between alcohol abuse, other substance abuse, sexual stigma and childhood and recent abuse. In a multivariable linear regression model, sexual stigma (beta = 0.17, 95% CI 0.03–0.32) and marriage to a woman (beta = −2.41 95% CI −4.74 to −0.09) were each associated with depression score.

**Conclusion:**

We found moderate to high levels of depression and substance abuse, and moderate levels of sexual stigma. These variables were highly inter-correlated and associated with an experience of trauma or abuse. Comprehensive mental health services are needed in this population to address these issues.

## Introduction

Men who have sex with men (MSM) are a marginalized population with particular health needs. They face many challenges, including stigma, social and legal discrimination, lack of social support and abuse [1–3]. These factors are intersecting and synergistic, and often result in social ostracization, decreased access to healthcare and, ultimately, worse health outcomes [3]. Fear of social and legal repercussions due to stigma may lead to nondisclosure of sexual risk behaviour and HIV serostatus. Nondisclosure has been linked to risky sexual behaviour, reduced medication adherence and poor engagement with healthcare [3,4].

These psychosocial challenges often lead to psychological distress, with MSM showing a higher prevalence of mental health conditions than other men [5–7]. Studies in the USA and Europe have shown that compared with general population men, MSM have up to a three-fold greater risk of major depression and dysthymia [5,6]. A study of mental disorders among MSM in South Africa found high levels of depression, alcohol and other substance abuse, and personality disorders [7]. Although limited in number, other studies among MSM in sub-Saharan Africa have found similar results [2,8].

Syndemic theory has been used to explain the reciprocal interaction between stigma, mental health, substance abuse and HIV risk [9,10]. Individual, social and structural factors combine to produce mutually reinforcing conditions that facilitate HIV transmission. Depression and substance abuse in MSM populations have been associated with unprotected intercourse, group sex and transactional sex [10–12]. Low resiliency and social support, often exacerbated by stigma, may reduce the ability of MSM to mitigate these factors and decrease their HIV risk. These intertwining factors have led to high HIV prevalence and incidence among MSM globally. In coastal Kenya, HIV prevalence among MSM was estimated at 24.5% [13]. A 2009 report by the Kenya National AIDS Control Council estimated that MSM accounted for 15.2% of new HIV infections in Kenya and over 20% of new infections on the coast [14].

Despite a growing body of evidence showing a high burden of mental health conditions and HIV infection among MSM in low-income and middle-income countries, there has generally not been a focus on surveillance, prevention or treatment programmes targeted at MSM in these settings [13,15]. For example, there are few MSM-specific psychosocial or HIV prevention or care interventions in Kenya, although small-scale interventions driven by external funding have recently begun [14,16,17]. Limited national and provincial budgets for most at-risk populations (MARPS), undertrained staff and an over-reliance on civil society organizations for these services have been cited as causes for this service gap [14,17].

Few studies have explored mental health conditions and associated psychosocial factors among MSM in sub-Saharan Africa. We could find no such studies conducted among Kenyan MSM [18]. The primary objective of this study was to determine the prevalence of depression and related factors, such as substance abuse, stigma, poor social support and abuse, among coastal Kenyan MSM. We hypothesized that these psychosocial factors would be correlated with each other and associated with depression. In part due to sexual and HIV-related stigma, we further hypothesized that HIV status would be correlated with depression in this population [2,5–8,19,20].

## Materials and methods

### Recruitment and data collection

This cross-sectional study was conducted between March and September 2014. Study participants were 112 self-identified MSM (defined as men who reported engaging in sex with a man within the past 3 months) drawn from ongoing HIV-seropositive and seronegative cohorts followed by the Key Populations Studies Group of the Kenya Medical Research Institute-Wellcome Trust Research Programme (KEMRI-WTRP) in Mtwapa, Kenya. The ongoing cohort studies collect data related to socio-demographic factors, sexual risk behaviour, substance use and current health problems and symptoms. The present study was used to pilot test questions for a more in-depth assessment of mental health and substance abuse. Participants were recruited when they presented for enrolment or follow-up appointments, at which time they were invited to complete the mental health questionnaire via audio computer-assisted self-interview (ACASI) in either English or Swahili. Of note, we have previously used ACASI successfully with this population during the ongoing cohort studies [21]. A dedicated ACASI monitor assisted with questionnaire administration as needed and was available to answer questions. Only one person refused to participate, of 113 approached.

### Measures

#### Depression

The Patient Health Questionnaire (PHQ) is a self-administered version of the Primary Care Evaluation of Mental Health Disorders (PRIME-MD), a tool developed to identify mental disorders in clinical settings [22,23]. The PHQ-9 is a depression-specific subset of the PHQ based on the nine DSM criteria for major depressive disorder [24,25]. The survey has been validated in North American and African populations [24–29]. It has been used in a wide variety of populations, including African HIV-seropositive adults [20,30–32], Kenyan populations [19,26,29] and MSM [33–35]. The PHQ-9 comprises nine questions rated on a 4-point Likert scale, which are summed for a total score from 0 to 27. The score can be interpreted using a diagnostic algorithm to generate a tentative diagnosis of major depressive disorder or other depressive disorder, as a continuous summary score or as a severity scale [24].

#### Alcohol abuse

Alcohol abuse was measured using the Alcohol Use Disorder Identification Test (AUDIT), a validated instrument developed for international settings [36]. The AUDIT has been used previously in stigmatized groups in East Africa (e.g. male sex workers in Mombasa) [37–39]. The survey has 10 questions scored 0–4 with potential scores ranging from 0 to 40. A score of at least 8 was considered indicative of hazardous drinking [36]. A categorical variable dividing AUDIT responses into four categories of severity was also used [36].

#### Substance abuse (nonalcohol, nontobacco)

Abuse of substances other than alcohol and tobacco (referred to as ‘other substance abuse’) was measured using an abridged 10-question version of the Drug Abuse Screening Test (DAST). The DAST-10 has been validated in a variety of populations, including those with mental illnesses [40,41]. The survey consists of 10 ‘Yes/No’ questions with scores ranging from 0 to 10. A validated cut-off score of at least 3 was used to identify harmful substance use [41]. A categorical severity scale was employed for descriptive purposes [42].

#### Sexual stigma

Sexual stigma was measured using an abridged version of the modified China MSM Stigma Scale by Logie *et al.* [43]. The scale measures two dimensions: perceived stigma, or the perception of social censure of homosexuality; and enacted stigma, the direct experience of homophobia. The modified measure replaced ‘because of your homosexuality’ with ‘because you have sex with men’ to make the terminology more reflective of participants’ context. It consists of 11 questions scored 0–3 (never; once or twice; a few times; many times). Potential scores ranged from 0 to 33, with a higher score representing greater perceived sexual stigma.

#### HIV stigma

Only respondents with known HIV-seropositive status completed the HIV stigma survey. We used two domains, negative self-image and disclosure concern factors, drawn from the abridged Berger HIV stigma scale by Kaai *et al.* [44,45]. Questions are graded on a four-point Likert scale (strongly disagree; disagree; agree; strongly agree). Potential scores ranged from 11 to 44, with a higher score indicating higher HIV stigma.

#### Perceived social support

The Medical Outcomes Survey-Social Support Survey (MOS-SSS) was used to measure perceived social support [46]. The survey covers four domains: emotional/informational support, tangible support, affectionate support, and positive social interaction, with one additional item dealing with social activities. The domains were collapsed to calculate one summary score, which was then transformed onto a 0–100 scale, with higher scores indicating higher perceived social support.

#### Abuse

Childhood abuse was measured using four questions drawn from the Childhood Experience of Care and Abuse (CECA), which has been validated in both clinical and community populations in the UK [47,48]. The CECA asks about four types of abuse as a child or a teenager: physical abuse at home, unwanted sexual experiences, forced or coerced sexual intercourse and upsetting sexual experiences with a related adult or authority figure. The assessment of recent (within the past year) abuse involved four items from the USAID HPI MSM Trauma Screening Tool [49]. This tool asks about four types of recent abuse: forced or coerced sex, physical abuse, emotional abuse and threats or intimidation. For both surveys, scores ranged from 0 to 4. We defined childhood abuse as any positive response on the CECA and recent abuse as any positive response on the USAID Screening Tool.

### Translation

Measures were independently translated from English to Swahili by two staff members fluent in both languages. The surveys were then back-translated into English to ensure they retained their original meaning. A validated Swahili translation was used for the PHQ-9 [29].

### Statistical analysis

Spearman’s rank correlation was used to calculate correlations between continuous variables, the rank biserial correlation (Somers’ D) for correlations between a continuous and a binary variable, and the phi coefficient (four-fold correlation) for correlations between two binary variables. Analyses involving HIV stigma were restricted to seropositive participants’ data only. Multi-collinearity was ruled out by examining variance inflation factors. Bivariable linear regression models were used to assess the association between individual variables and PHQ-9 depression score. Sociodemographic and behavioural factors were identified as potential confounders on the basis of existing literature [6,11,39]. Variables with a significance level of 0.20 or less were included in the multivariable linear regression model. Robust regression was used to protect against heteroscedasticity.

### Ethics statement

The ongoing cohort studies are approved by the ethical review boards of the Kenya Medical Research Institute and the University of Washington. All men gave written informed consent to participate in the ongoing cohorts and verbal consent to participate in the pilot test of the mental health ACASI. Participants were asked to debrief with a counsellor after completing the survey, in order to identify any problems or questions that had been upsetting.

## Results

One HIV-positive participant did not have HIV stigma data due to having erroneously selected HIV-negative status on the survey. This participant was excluded from HIV stigma analysis. For 17 participants who completed the ACASI on a different date from their scheduled study visit, data on transactional sex of sexual partners were imported from the nearest clinic visit (within 30 days). Table 1 presents descriptive characteristics of the study population as well as prevalence and summary statistics for psychosocial characteristics. The majority of participants were young (median age 26 years), unmarried (92.9%), had at least some secondary education (66.1%), earned less than 10 000 Kenyan Shillings ($109 US) per month (73.2%) and had sex with men exclusively (62.5%). Forty-nine percent of respondents were HIV-positive and 31.3% had engaged in sex work in the past 3 months. Religious affiliation was divided between none (30.4%), Catholic (29.5%), Protestant (17.9%) and Muslim (22.3%).

Roughly one-third of respondents met criteria for either major depressive disorder (16.1%, 95% CI 9.8–24.2) or other depressive disorder (15.2%, 95% CI 9.1–23.2), and 42.0% had moderate to severe depressive symptoms. Further, 75.0% of all respondents reported anhedonia, 75.9% depressed mood and 38.4% ideation of suicide or self-harm more than half the days in the last 2 weeks. Forty-five percent engaged in hazardous or harmful drinking behaviour (95% CI 35.2–54.3) and 59.8% in harmful use of other substances (95% CI 50.1–69.0). Sexual and HIV stigma were moderate, with median scores of 11 (range 0–33) and 25 (range 17–40), respectively. Perceived social support had a median score of 68 (range 19–95). The majority of participants reported some form of abuse within the past year (67.0%) and some form of abuse in childhood (77.7%). Roughly one-half of respondents (47.3%) reported either forced sexual intercourse or an upsetting sexual experience with a related adult or authority figure before the age of 17, with 22.3% reporting both. Moderate to high Cronbach’s alphas (0.7–0.9) suggest good internal consistency reliability of the survey items within our population.

Table 2 presents bivariable associations between regression model predictors using Spearman’s correlation coefficients, rank biserial correlation coefficients and phi coefficients. There were significant correlations between alcohol abuse, other substance abuse, sexual stigma, childhood abuse and recent abuse. For the most part, these relationships were of moderate strength, with correlation coefficients ranging from 0.27 to 0.64. HIV status, HIV stigma and perceived social support were not significantly correlated with any other measures.

Table 3 presents the unadjusted and adjusted linear regression analysis of factors associated with depressive symptoms in this population. Continuous summary scores were used for PHQ-9 (outcome), and for AUDIT, DAST-10, sexual stigma, HIV stigma and perceived social support (predictors). HIV status and trauma were analysed as binary predictors of depression. In bivariable analysis, higher scores for alcohol abuse were associated with higher PHQ-9 scores (beta = 0.19, 95% CI 0.07–0.31), as were higher sexual stigma scores (beta = 0.31, 95% CI 0.19–0.44). Childhood and recent abuse were both positively associated with higher PHQ-9 scores (beta = 5.09, 95% CI 2.67–7.51, and beta = 4.77, 95% CI 2.48–7.07, respectively). The association between higher other substance abuse scores and higher PHQ-9 scores was of borderline significance (beta = 0.43, 95% CI −0.01 to 0.86). Socioeconomic status (estimated by personal asset count), marital status and sex of sexual partners were included in the multivariable model, as they had associations with PHQ-9 score that met the *P* value less than 0.2 criteria for inclusion. In the multivariable model, only two factors remained significantly associated with depressive symptoms: higher sexual stigma scores were associated with higher PHQ-9 scores (beta = 0.17, 95% CI 0.03–0.32), while marriage to a woman was associated with lower PHQ-9 scores (beta = −2.41, 95% CI −4.74 to −0.09).

## Discussion

This study is the first we are aware of to explore depressive symptoms and related psychosocial factors among Kenyan MSM. We found high levels of alcohol and other substance abuse and of both childhood and recent abuse, as well as moderate levels of sexual stigma. These factors were shown to be highly intercorrelated and associated with PHQ-9 score, our measure of depressive symptoms. This is in keeping with other research, which has shown that adverse childhood events can lead to an increased risk of depression and substance abuse conditions as well as abuse later in life [50]. There is also a wealth of research showing associations between violence and alcohol and other substance abuse [51].

Participants exhibited high levels of depression (i.e. almost one-third met criteria for major or other depressive disorder), considerably higher than the reported national prevalence of depression in Kenya of 6.7% [52]. MSM in the USA, Netherlands, South Africa and Tanzania have also displayed a similarly increased risk of depression [2,5–8]. Although many participants did not meet the diagnostic criteria for a depressive disorder, the majority still suffered from depressive symptomology. Many participants gave positive responses to specific PHQ-9 questions, such as anhedonia and depressed mood. Due to high reports of ideation of suicide or self-harm, the research clinic provided additional training to clinicians and counsellors on how to screen for and manage suicidality.

We have previously documented both high HIV prevalence and incidence among MSM participating in this research cohort [13,17]. Contrary to our hypotheses, HIV status and HIV stigma were not associated with depression, nor were they associated with substance abuse or any of the other psychosocial factors we measured. This result was unexpected, as prior research has found significant associations between HIV status, stigma and mental health conditions [19,20,53], although a recent study in India also found no such connection [54].

Although further research will be necessary to fully understand these results, we hypothesize that sexual stigma may have a larger impact than HIV stigma among Kenyan MSM due to greater social censure related to same-sex behaviour. Antiretroviral therapy has transformed AIDS from an often visible death sentence to a treatable chronic condition, and destigmatization efforts have been included in a number of HIV/AIDS programmes in Kenya and across East Africa [55]. There have not been corresponding antihomophobia or otherwise MSM-focused destigmatization campaigns. Criminalization of same-sex practices and unions have either remained intact or become harsher, as was the case with the Ugandan Anti-Homosexuality Act of 2014. In Kenya, same-sex activities can carry jail terms of up to 14 years [56]. Although not officially prohibited by law, same-sex marriage is not practiced or accepted. Rumours of a same-sex marriage led to an attack on the Mtwapa research clinic in 2010, which resulted in a temporary closure [57].

Of particular concern were high rates of sexual abuse. Almost 30% of participants reported forced or coerced sex in the past year. For childhood abuse, roughly one-half of respondents reported sexual abuse before the age of 17. This is significantly higher than Kenyan national averages according to a UNICEF report of childhood abuse and neglect [58]. The report found that 17.5% of male respondents self-reported any sexual abuse prior to age 18, of whom 1.4% reported physically forced sex. Childhood abuse is associated with a wide range of negative outcomes. In a sample of general population Kenyans, childhood abuse and neglect were found to be significant predictors of criminal tendencies, depressive symptoms and borderline personality symptoms [59]. As a highly prevalent and strong predictor of mental health conditions, counselling for victims of childhood abuse and forced sex should be an integral component of MSM-focused interventions.

In the multivariable model, after adjusting for sociodemographic, behavioural and psychosocial factors, only two factors remained statistically significant: being married to a woman and sexual stigma. Being married to a woman was protective against depression. A stable relationship could be a source of social support and positive reinforcement, although, counter-intuitively, perceived social support as an independent factor was not associated with depression. Marriage to a woman can also help MSM hide their sexual orientation and be more socially accepted. Marital status was not associated with sexual or HIV stigma, income or socioeconomic status, all of which were anticipated pathways through which marital status might mediate depressive symptoms. Further study into sociodemographic and psychosocial differences between married and unmarried MSM is necessary to fully understand these findings.

Sexual stigma was a risk factor for depression in this population. There has been considerable research into the association between stigma and depression. Studies have shown that high levels of sexual stigma can lead to internalized homophobia, low resiliency and poor social support, all of which are risk factors for mental health conditions [43,60]. The minority stress model has been used to describe this relationship, arguing that stigma and discrimination against sexual minorities acts as a source of sustained stress, which can result in negative health outcomes, including depression [60]. Resiliency and social support, which can act as protective mediators between stress and health outcomes, are often reduced for sexual minorities, resulting in greater negative impacts on health. MSM in Kenya are often isolated due to the fear of negative consequences of coming out, although community-based organizations and other civil society groups have begun to provide additional social support and training to increase resiliency and other skills.

Our study had some limitations, including a limited sample size and sampling frame, which may restrict generalizability to other MSM groups. Participants were drawn from those attending the clinic for routine follow-up in ongoing cohort studies with monthly or quarterly visits. MSM who are willing to self-identify as such and participate in this research may be different from MSM who choose not to. It is possible that our results underestimate the true prevalence and burden of these conditions, as health-seeking behaviour and research participation are likely to be negatively associated with mental health and substance abuse conditions. The cross-sectional nature of this study also limits inference of causality (e.g. substance abuse leading to depression). Additional, longitudinal data collection will be necessary to determine causal pathways.

Psychosocial measures (e.g. feelings of unhappiness) introduce a level of subjectivity, as they depend on self-report of subjective experiences, which may result in some misclassification error. Well validated surveys were chosen to reduce such error. Moderate to high Cronbach’s alphas show good internal reliability of the survey items within our population. ACASI was used for data capture, as it has been shown to reduce response bias and underreporting due to perceived social stigma in studies among stigmatized populations, including among MSM sex workers in this same cohort [21]. The audio component has also been shown to reduce the negative effects of illiteracy on data collection [61,62].

In response to our findings, we have begun integrating regular mental health and substance abuse screening and counselling programmes at the study research clinic. Mental health issues are generally not emphasized in training of HIV counsellors in Kenya [17], and we have had to provide supplemental training to our clinic staff. We are also working to export use of the screening tool to other MSM cohorts in Kenya. Although small steps, we hope that these efforts represent a beginning for targeted mental health programming for MSM in Kenya.

More generally, we recommend that mental health screening and treatment or referral should be integrated into ongoing HIV prevention and treatment programmes. In particular, these programmes should seek to address alcohol and substance abuse, childhood abuse and sexual stigma, in addition to HIV stigma as many now do. Existing mental health and substance abuse programmes should be expanded and better supported, in order to respond to the dearth of services for both MSM and the general population, regardless of HIV status. National and local policy should be adapted to prioritize these challenges.

## Figures and Tables

**Table 1:** Demographic and psychosocial characteristics and internal test consistency (*n* = 112).

Characteristic	*n* (%) or med (IQR)	Cronbach’s alpha
Age (years)	26 (23.5–30)	
Earned income		
≤KSh 2000	8 (7.1%)	
KSh 2000–5000	28 (25.0%)	
KSh 5000–9000	46 (41.0%)	
KSh 10 000–19 000	23 (20.5%)	
≥KSh 20 000	7 (6.3%)	
Personal assets		
Private water source	22 (19.6%)	
Private toilet	24 (21.4%)	
Electricity	72 (64.3%)	
Radio	85 (75.9%)	
Television	54 (48.2%)	
Mobile phone	78 (69.6%)	
Median asset count	3 (1.5–4)	
Religion		
None	34 (30.4%)	
Catholic	33 (29.5%)	
Protestant	20 (17.9%)	
Muslim	25 (22.3%)	
Marital status		
Single	97 (86.6%)	
Married (to a woman)	8 (7.1%)	
Divorced/other	7 (6.3%)	
Education		
Primary (<8 years)	13 (11.6%)	
Primary (8 years)	25 (22.3%)	
Secondary	56 (50.0%)	
Tertiary	18 (16.1%)	
Sex work (in the last 3 months)		
Yes	35 (31.3%)	
Sex of sexual partners		
Men only	70 (62.5%)	
Women only	5 (4.5%)	
Women and men	37 (33.0%)	
Depression		0.86
Minimal (0–4)	26 (23.2%)	
Mild (5–9)	39 (34.8%)	
Moderate (10–14)	21 (18.8%)	
Mod. severe (15–19)	20 (17.9%)	
Severe (≥ 20)	6 (5.4%)	
Major depressive disorder	18 (16.1%)	
Other depressive disorder	17 (15.2%)	
Alcohol abuse		0.87
Zone I (0–7)	62 (55.4%)	
Zone II (8–15)	28 (55.4%)	
Zone III (16–19)	6 (5.4%)	
Zone IIII (20–40)	16 (14.3%)	
Alcohol abuse (AUDIT ≥8)	50 (44.6%)	
Substance abuse		0.78
None (0)	2 (1.8%)	
Low (1–2)	43 (38.4%)	
Moderate (3–5)	42 (37.5%)	
Substantial (6–8)	15 (13.4%)	
Severe (9–10)	10 (8.9%)	
Substance abuse (DAST-10 ≥3)	67 (59.8%)	
Sexual stigma (range: 0–33)	11 (6–17)	0.85
HIV stigma^a^ (range: 0–40)	25 (23–29)	0.61
Perceived social support (range: 0–100)	68 (57–78.5)	0.93
Childhood abuse (any)	87 (77.7%)	0.70
Physical abuse at home	75 (67.0%)	
Unwanted sexual experience	50 (44.6%)	
Forced or coerced intercourse	40 (35.7%)	
Upsetting sexual experience with family member or authority figure	38 (33.9%)	
Recent abuse (any)	75 (66.9%)	0.76
Forced or coerced sex	33 (29.5%)	
Physical abuse	43 (38.4%)	
Emotional abuse	58 (52.0%)	
Threats or intimidation	57 (50.9%)	
HIV-positive (yes)	55 (49.1%)	

a Among seropositive participants only. One participant had missing data for HIV stigma due to an error entering his HIV status.

**Table 2:** Correlation between regression model predictors.

	1	2	3	4	5	6	7
1. Alcohol abuse	–						
2. Other substance abuse	0.40^b^ **	–					
3. Sexual stigma	0.41^b^ **	0.27^b^ *	–				
4. HIV stigma^a^	−0.06^b^	−0.14^b^	0.01^b^	–			
5. Social support	0.01^b^	0.05^b^	0.02^b^	0.13^b^	–		
6. Childhood abuse	0.36^c^ *	0.43^c^ **	0.56^c^ **	−0.04^c^	0.03^c^	–	
7. Recent abuse	0.48^c^ **	0.44^c^ **	0.64^c^ **	0.11^c^	0.10^c^	0.64^d^ **	–
8. HIV status	0.01^c^	0.04^c^	0.21^c^	–	0.17^c^	0.03^d^	0.01^d^

* *P* <0.01.

** *P* <0.0001.

a Seropositives only.

b Spearman’s rho.

c Rank biserial correlation coefficient.

d Phi coefficient (sqrt(X^2^/n)).

**Table 3:** Linear regression on Patient Health Questionnaire score.

	Unadjusted beta	*P*	Adjusted beta	*P*
Age	0.02 (−0.23 to 0.26)	0.89		
Earned income		0.69		
≤KSh 2000	Reference	–		
KSh 2000–5000	−1.32 (−4.76 to 2.12)	0.45		
KSh 5000–9000	0.25 (−3.22 to 3.72)	0.89		
KSh 10 000–19 000	0.40 (−3.25 to 4.05)	0.83		
≥KSh 20 000	1.46 (−6.28 to 9.20)	0.71		
Personal assets (median asset count)	0.54 (−0.15 to 1.23)	0.13	0.56 (−0.17 to 1.30)	0.13
Religion		0.40		
None	Reference	–		
Catholic	0.71 (−2.18 to 3.61)	0.63		
Protestant	−1.64 (−4.71 to 1.44)	0.29		
Muslim	−1.38 (−4.47 to 1.69)	0.37		
Marital status		0.05		
Single	Reference	–	Reference	–
Married (to a woman)	−3.70 (−6.76 to −0.65)	0.02	−2.41 (−4.74 to −0.09)	0.04
Divorced/Other	−1.86 (−6.07 to 2.35)	0.38	−1.60 (−5.28 to 2.08)	0.39
Education		0.91		
Primary (<8 years)	Reference	–		
Primary (8 years)	0.48 (−3.56 to 4.52)	0.81		
Secondary	−0.16 (−3.72 to 3.40)	0.93		
Tertiary	1.06 (−3.81 to 5.92)	0.67		
Sex work (in the last 3 months)				
Yes	1.04 (−1.33 to 3.42)	0.39		
Sex of sexual partners		0.14		
Men only	Reference	–	Reference	–
Women only	−1.19 (−8.71 to 6.34)	0.76	2.40 (−4.29 to 9.09)	0.48
Women and men	−2.23 (−4.45 to −0.003)	0.05	−1.28 (−3.30 to 0.73)	0.21
Alcohol abuse	0.19 (0.07 to 0.31)	0.002	0.06 (−0.07 to 0.20)	0.36
Substance abuse	0.43 (−0.01 to 0.86)	0.05	−0.05 (−0.52 to 0.42)	0.84
Sexual stigma	0.31 (0.19 to 0.44)	<0.001	0.17 (0.03 to 0.32)	0.02
HIV stigma^a^	−0.01 (−0.32 to 0.15)	0.48		
Perceived social support	0.01 (−0.06 to 0.08)	0.70		
Childhood abuse (yes)	5.09 (2.67 to 7.51)	<0.001	2.63 (−0.72 to 5.98)	0.12
Recent abuse (yes)	4.77 (2.48 to 7.07)	<0.001	1.42 (−1.92 to 4.78)	0.40
HIV-positive status (yes)	−0.35 (−2.55 to 1.85)	0.90		

a Seropositives only.
